# The use of matrine to inhibit osteosarcoma cell proliferation via the regulation of the MAPK/ERK signaling pathway

**DOI:** 10.3389/fonc.2024.1338811

**Published:** 2024-08-05

**Authors:** Xincheng Huang, Jun Zeng, Siyuan Ruan, Zhuolin Lei, Jingyuan Zhang, Hong Cao

**Affiliations:** ^1^ Department of Traumatic Orthopedics, Renmin Hospital, Hubei University of Medicine, Shiyan, China; ^2^ Department of Anesthesiology, Shiyan People’s Hospital, Shiyan, China

**Keywords:** matrine, osteosarcoma, MAPK/ERK signaling, antitumor effects, pathway

## Abstract

**Background:**

Matrine is an alkaloid extracted from Sophorus beans of the legume family, and it has significant effects and a variety of pharmacological activities. Osteosarcoma(OS) is a common malignant bone tumor that is characterized by high incidence and rapid progression. There have been some preliminary studies on the therapeutic effect of matrine on OS, but the specific mechanism remains unclear.

**Objective:**

The aim of this study was to investigate the antitumor effect of matrine on HOS cells and the underlying molecular mechanism.

**Methods:**

The effects of matrine on the proliferation, apoptosis and cell cycle progression of HOS cells were determined by CCK-8 assay, TUNEL assay and flow cytometry *in vitro*. Wound healing and Transwell invasion assays were used to observe the effect of matrine on the migration and invasion of HOS cells. The mechanism underlying the antitumor effect of matrine on HOS cells was investigated by Western blotting.

**Results:**

Matrine significantly inhibited HOS cell proliferation, promoted HOS cell apoptosis, and arrested HOS cells in the G1 phase of the cell cycle. Both wound healing and Transwell invasion assays showed that matrine inhibited HOS cell migration and invasion. Western blotting results showed that matrine inhibited the activation of the MAPK/ERK signaling pathway. We found that matrine also downregulated Bcl-2 expression, which may be related to protein synthesis inhibition.

**Conclusion:**

Matrine can inhibit the proliferation of HOS cells, arrest HOS cells in the G1 phase, and promote HOS cell apoptosis through the MAPK/ERK signaling pathway.

## 1 Introduction

Osteosarcoma (OS) is one of the most common primary malignant bone tumors. OS is a tumor that is derived from osteogenic mesenchymal stem cells; OS mainly affects the fastest growing bone structures in children and adolescents, such as the distal femur, proximal tibia and proximal humerus metaphysis region (1), and it occurs less frequently in the skull, jaw, and pelvis (2). Persistent local pain and venous distention are the main clinical manifestations of OS. When patients develop generalized cachexia, it seriously threatens their lives. Recent IARC data indicate that primary malignant bone tumors are a common type of cancer in children and adolescents. Primary malignant bone tumors are estimated to account for 3% to 5% of cancer cases in children and adolescents, but these tumors are rare in adults, accounting for 1% of all cancer cases in adults (3, 4). The initial symptoms are often local swelling and pain, and these tumors are not easily diagnosed and taken seriously. It is often difficult to effectively treat these tumors due to their small size and the limited treatment methods. Moreover, OS is prone to lung metastasis, there is a lack of effective therapeutic targets, and it easily develops drug resistance. As a result, OS is associated with high disability and mortality rates, and it is difficult to cure. Although neoadjuvant chemotherapy has been a standard treatment in clinical practice in recent years, the prognosis of patients with OS is still unsatisfactory (5). Therefore, the search for more effective treatment methods to improve patient survival rates has become the focus of scientific research and clinical work. In recent years, with the development of the economy, the application of traditional Chinese medicine in new drug development and treatment has become increasingly extensive. The role of traditional Chinese medicine in treatment has become increasingly prominent. For example, in ovarian cancer, pancreatic cancer, colon cancer, gastric cancer and other cancers, berberine can exert anticancer effects by inhibiting cell proliferation (6–8). Ginsenoside can play a role in treating colon cancer, non-small cell lung cancer, breast cancer and other cancers by promoting tumor cell apoptosis (9, 10). Sanguinarine (SANG), a naturally isolated plant alkaloidal agent, possesses chemo-preventive effects. Several studies have revealed that SANG impedes tumor metastasis and development by disrupting a wide range of cell signaling pathways and its molecular targets, such as BCL-2, MAPKs, Akt, NF-κB, ROS, and microRNAs (miRNAs) (11). Similarly, matrine, an alkaloid extracted from the traditional Chinese medicine *Sophora flavescens* Aiton, has been shown to have potential anticancer properties (12, 13). Matrine is known to exert a variety of pharmacological effects, including anti-inflammatory, antiallergic, antiviral, antifibrotic, and cardiovascular protective effects. Furthermore, matrine has demonstrated significant potential for use in cancer treatment due to its ability to accelerate cell apoptosis, inhibit tumor cell growth and proliferation, induce cell cycle arrest, suppress cancer metastasis and invasion, inhibit angiogenesis, promote autophagy, reverse multidrug resistance, and inhibit cell differentiation (12). The mitogen-activated protein kinase/extracellular regulated protein kinase (MAPK/ERK) pathway plays a key role in cancer progression (14). The MAPK/ERK signaling pathway, with its intricate cascade and broad spectrum of biological functions, continues to be a significant focus for understanding cellular processes and diseases, especially cancer. Recent literature not only provides insights into the core mechanism but also explores the pathological significance and interactions of this pathway, making it a promising direction for future research and therapeutic interventions. The aim of this study was to investigate how matrine affects the proliferation of HOS cells by regulating the MAPK/ERK signaling pathway. The results show that matrine has the potential to be used in the treatment of OS.

## 2 Materials and methods

### 2.1 Materials

Matrine was obtained from the Hubei Key Laboratory of Wu, dang Local Chinese Medicine. HOS cells were obtained from the Shanghai Institute of Biochemistry and Cell Biology, Chinese Academy of Sciences (Shanghai, China). MEM, 1640 medium, 1% penicillin–streptomycin solution, and trypsin solution (0.25% trypsin) were purchased from Procell Life Science & Technology Co., Ltd. Fetal bovine serum (FBS) (10%) was purchased from ExCell Bio. CCK-8 was purchased from MCE. Phenylmethanesulfonylfluoride (PMSF) was purchased from Aladdin Co., Ltd. (Shanghai). PD98059, RIPA lysis buffer, a bicinchoninic acid (BCA) protein assay kit, propidium iodide (PI), and dihydrochloride (DAPI) were purchased from Beyotime Biotechnology Co., Ltd. (China). Tetramethylethylenediamine (TEMED) was purchased from Sinopharm Chemical Reagent Co., Ltd. Annexin V-FITC and PI were purchased from Vazyme Biotech Co., Ltd. Antifade Mountant Medium Biogradetech was purchased from SouthernBiotech. Poly (vinylidene fluoride) (PVDF) membranes were purchased from Millipore. A rabbit anti-Bcl2 polyclonal antibody and a rabbit anti-BAX monoclonal antibody were purchased from Bioss Biotechnology Co., Ltd.

### 2.2 Cell culture and viability assay

HOS cells were seeded in MEM supplemented with 10% FBS and 1% penicillium-streptomycin at 37°C in 5% carbon dioxide. When the confluence of the cells reached 80%, 1-2 ml of 0.25% trypsin was added to digest the cells. After digestion, the trypsin was quickly discarded, and the cells were passaged at a ratio of 1:3. Cells in the exponential phase of growth were selected and seeded in 96-well plates (3×10^3^ cells/well) to establish a blank cell group. The cells in both groups were incubated for 24 h at 37°C in 5% carbon dioxide. Then, the cells were divided into three groups: one group of culture dishes was treated with 5 µg/ml matrine, another group was treated with PD98059 and matrine, and the remaining group of untreated cells served as the control group. All the cells were incubated in fresh medium for 72 h. Ten microliters of CCK-8 solution was added to each well, and the incubation was continued for 4 h. The optical density (OD) of the cells in each well was determined at a wavelength of 450 nm using an enzyme-linked immunosorbent assay (Molecular Devices, USA).

### 2.3 Cell apoptosis assay

Passaged cells in the exponential phase of growth were seeded in 6-well plates (3×10^5^ cells/well) and incubated for 24 h at 37°C in 5% carbon dioxide. After the cells were plated, the plates were divided into three groups: one group was incubated with 5 µg/ml matrine, and the other group was treated with D98059 and matrine, and untreated cells were used as the control group. All of these cells were incubated in fresh medium for 72 h. After 72 hours, the cells were collected by trypsin digestion, treated with 10 μL of RNase, and incubated at 37°C for 30 minutes. Then, 10 μL PI was added, and the cells were stained at 4°C in the dark for 30 minutes. The apoptosis rate was determined by flow cytometry.

### 2.4 Wound healing assay

Cells were seeded in 6-well plates (5×10^5^cells/well) and incubated at 37°C in 5% CO_2_ for 24 h. Wounds were created in monolayers of HOS cells with sterile pipette tips. The cells that were wounded by the pipette tip were aspirated from the culture plate, and the remaining cells were treated with fresh serum-free medium supplemented with different concentrations of matrine for 24 h. The change in the width of the wound at the same site was observed at 0 h and 24 h.

### 2.5 Cell migration and invasion assay

In the invasion assay, HOS cells were resuspended in serum-free 1640 medium, diluted to a concentration of 2×10^4^ cells/ml in serum-free MEM, and set aside. Then, 800 µl of medium supplemented with was added to the lower chamber of the Transwell plate. Next, 100 µl of Matrigel (final concentration of 1 mg/ml) was added to the upper chamber of the Transwell plate and allowed to solidify at 37°C for 4-5 hours to form a Matrigel membrane. After the Matrigel had formed a gel, 200 µl of a suspension of matrine-treated cells was added to the upper chamber of the Transwell plate and incubated at 37°C in a 5% CO_2_ incubator for 24 h. The cells were then fixed with 70% cold ethanol for 1 h, stained with 0.5% crystal violet solution and incubated at room temperature for 20 min. After washing with PBS, any cells that had not crossed into the lower chamber from the upper chamber were removed. The cells were observed with an inverted phase contrast microscope (Olympus, Japan). The migration assay procedure was the same as that for the invasion assay, except that Matrigel was not used.

### 2.6 Western blotting analysis

After harvesting the treated cells, 100 µl of lysis buffer containing PMSF was added to each well, and the cells were lysed for 30 min on ice. The plates were centrifuged at 12000 rpm for 5 min at 4°C, and the supernatants were carefully collected. Protein concentrations were analyzed using the BCA kit according to the manufacturer’s instructions. The OD value was determined using an OS-3022A microplate reader. The linear regression equation was calculated from the standard protein concentrations and the corresponding OD values, and the sample protein concentrations were calculated using the regression equation based on the OD values of the protein samples. Protein loading buffer was then added to the protein solutions, and the proteins were denatured for 10 min at 95°C. The proteins (40 µg) were then loaded onto 10% gels, separated by SDS–PAGE, and transferred to PVDF membranes. The membranes were then blocked with 5% skim milk for 2 h and incubated with the appropriate primary antibody (1:1000) overnight at 4°C on a shaker. The PVDF membranes were washed five times with TBST, and then secondary antibodies (1:10 000) were added and incubated for 2 h at room temperature on a shaker. Finally, the ECL kit (Applygen, China) was used for development according to the supplier’s instructions, and the gray values on the film were analyzed using BandScan.

### 2.7 Terminal deoxynucleotidyl transferase-mediated dUTP nick end labeling assay

Treated cells were collected and fixed with 4% paraformaldehyde at pH 7.4 for 15 min, followed by three washes with PBS to remove residual fixative. To ensure the penetration of subsequent reagents, the samples were treated with 20 μg/ml proteinase K solution for 20 min. The slides were then coated with 1× equilibration buffer and incubated at room temperature for 15 min in preparation for further labeling. After equilibration, TdT incubation buffer was added and incubated for 60 min at 37°C. To clearly visualize the nuclei, the nuclei were stained with DAPI for 5 min and subsequently washed to remove excess dye. Finally, for long-term preservation and observation, we sealed the slides with mounting solution containing anti-fluorescence quenching agents, and images were acquired under a fluorescence microscope.

### 2.8 Statistical analysis

The data were calculated based on triplicate experiments and are expressed as the mean ± SD. The data were analyzed using SPSS 25.0 statistical software, and cell counting was performed with ImageJ software. The graphs were generated with GraphPad Prism 8.0. Comparisons between two or more groups were performed by Student’s *t* test or one-way analysis of variance. *P* <0.05 was considered to be statistically significant.

## 3 Results

### 3.1 Matrine inhibited HOS cell activity and proliferation

**Figure 1:**
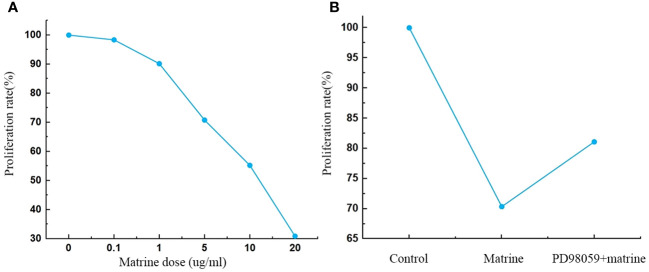
**(A, B)** Matrine inhibited HOS cell proliferation. The proliferation of HOS cells was decreased after matrine treatment in a dose-dependent manner. HOS cell proliferation was enhanced after pathway inhibitor treatment.

HOS cells were treated with different concentrations of matrine for 72 h *in vitro*, and the effect of matrine on cell viability was analyzed by CCK8 assay. **Figure 1A** shows that matrine significantly inhibited the activity of HOS cells, and the inhibitory effect was enhanced with increasing matrine concentration (P < 0.01). After the addition of an MAPK/ERK pathway inhibitor (PD98059), the cell activity was enhanced.

### 3.2 Matrine induced HOS cell apoptosis and cell cycle arrest in the G1 phase

**Figure 2:**
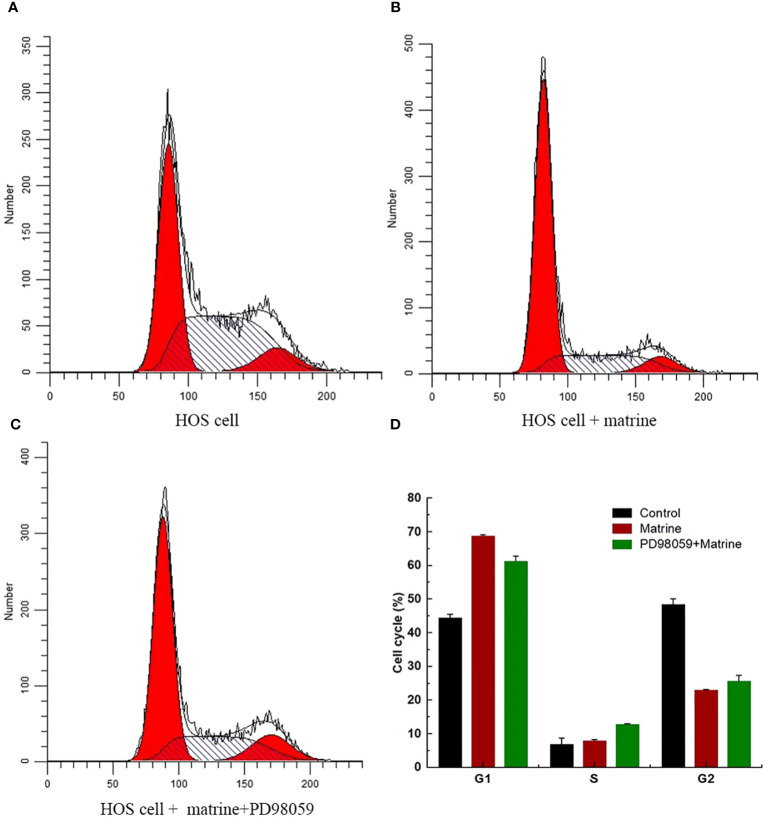
**(A–D)** Flow cytometry analysis of matrine-induced arrest of HOS cells in the G1 phase.

To determine the effect of matrine on HOS cell cycle progression, flow cytometry was performed, and the cell cycle progression of HOS cells was determined, as shown in the (**Figures 2A–D**). Matrine caused a significant increase in the number of cells in the G1 phase. These results indicated that matrine increased HOS cell apoptosis and induced cell cycle arrest in the G1 phase.

### 3.3 Matrine Inhibits HOS Cell Migration and Invasion

**Figure 3:**
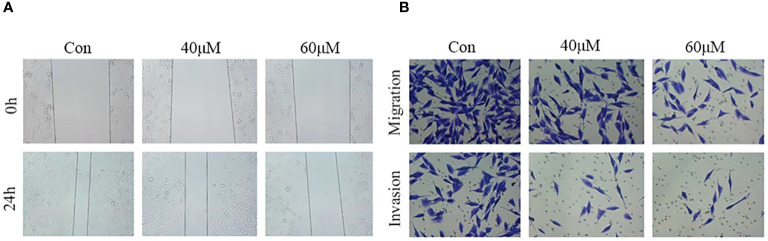
Matrine inhibits the migration and invasion of HOS cells. **(A)** In the wound healing assay, the wound area was significantly larger in the matrine treatment group than in the control group. **(B)** The migration and invasion ability of HOS cells was significantly inhibited after treatment with different concentrations of matrine.

Cell migration and invasion are hallmarks of tumor progression. To assess the effect of matrine on HOS cell migration and invasion, wound healing and Transwell assays were performed after treating the cells with different concentrations of matrine for 24 hours. The results of the wound healing assay (**Figure 3A**) showed that the wound area was significantly larger in the matrine treatment group than in the control group. The Transwell results (**Figure 3B**) showed that the number of migrating and invading HOS cells was significantly lower in the 40 and 60 µM matrine treatment groups than in the control group. These results indicated that matrine inhibited the migration and invasion of HOS cells in a concentration-dependent manner.

### 3.4 Matrine regulated the expression of MAPK/ERK pathway-related proteins in HOS cells

**Figure 4:**
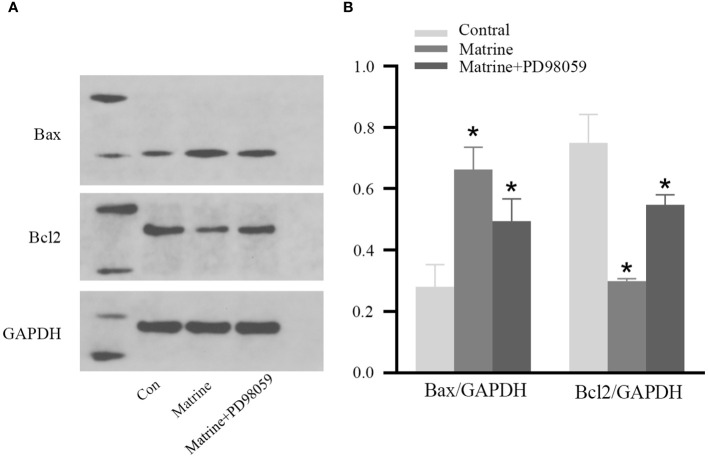
Matrine inhibited activation of the MAPK/ERK pathway in HOS cells. IHC results of HOS cells. **(A, B)** Matrine downregulated the expression and phosphorylation of Bcl-2 and upregulated the expression and phosphorylation of Bax in HOS cells. *P<0.05.

To further investigate the mechanism underlying the antitumor effects of matrine on HOS cells, the expression level of MAPK/ERK signaling pathway components was determined. Western blotting was used to measure the expression and phosphorylation of Bax and Bcl-2, the key regulatory proteins in the pathway. As shown in **Figures 4A, B**, matrine and PD98059 had similar effects. The expression and phosphorylation of Bcl-2 were decreased, while the expression and phosphorylation of Bax were increased in a dose-dependent manner. These data suggest that matrine may inhibited the activation of the MAPK/ERK signaling pathway in HOS cells.

### 3.5 Matrine induced apoptosis in HOS cells

**Figure 5:**
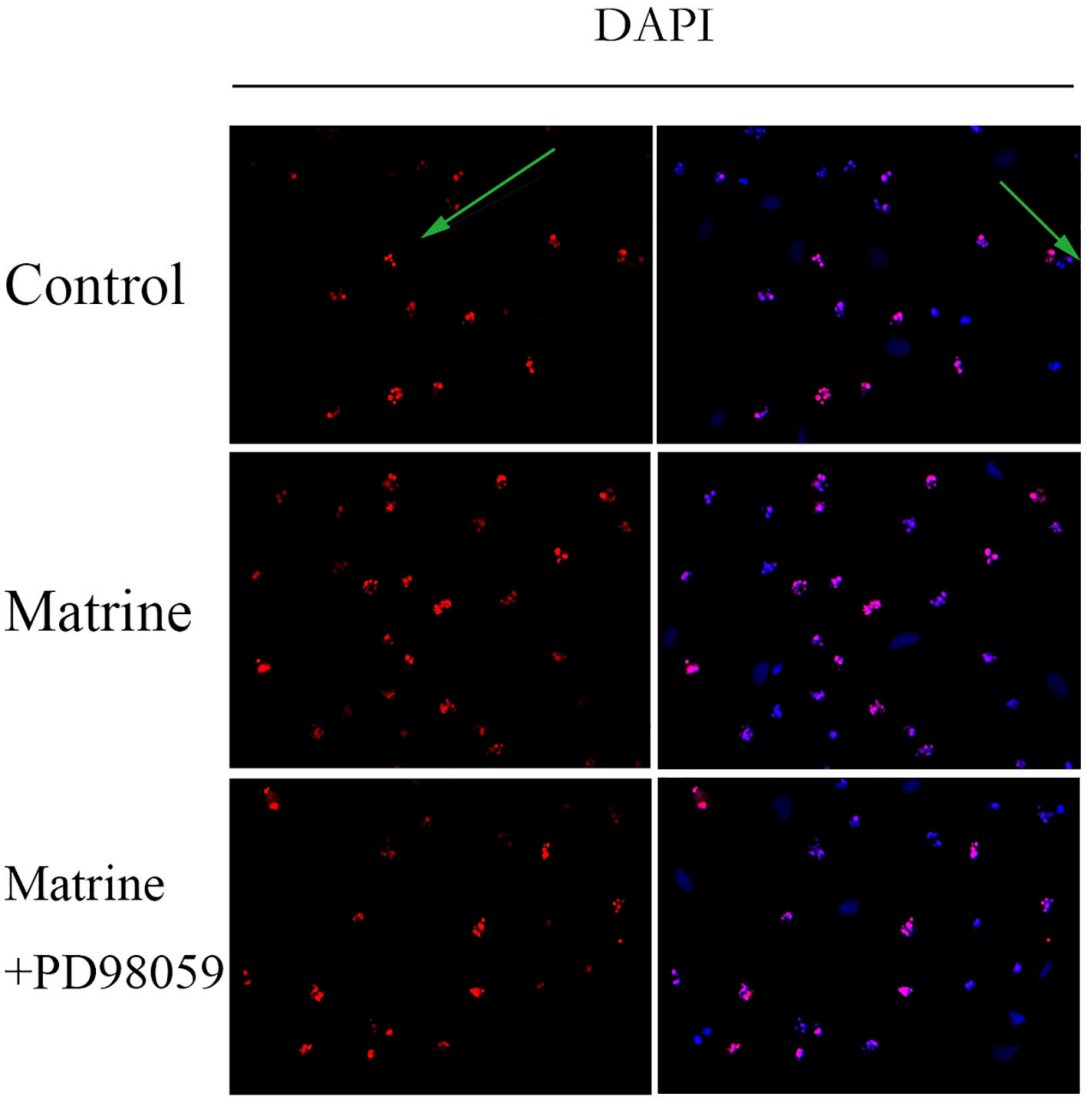
Matrine induces apoptosis in human OS cells. HOS cells stained with DAPI reagent and observed under a fluorescence microscope (400×) exhibited morphological characteristics of early apoptotic cells. The apoptotic cells emitted red fluorescence, and the nuclei emitted blue fluorescence. Matrine-treated HOS cells were stained with DAPI reagent and observed under a fluorescence microscope (400x), and apoptotic bodies began to appear. HOS cells treated with matrine and PD98059 were stained with DAPI reagent and observed under a fluorescence microscope (400x). The apoptosis rate of the treated cells was much higher than that of the control cells, and most of the apoptotic cells were in the early stage of apoptosis.

To investigate the effect of matrine on HOS cell apoptosis, a TUNEL assay was performed. DAPI staining showed that matrine-treated OS cells exhibited morphological features of early apoptotic cells, such as bright nuclear condensation or debris (**Figure 5**). At higher matrine concentrations, apoptotic bodies began to appear. We also found that higher matrine concentrations increased the number of late apoptotic cells. The greatest cell death occurred within 48 hours after drug treatment (data not shown). Next, we added PD98059, a pathway inhibitor, and found that matrine exerted effects that were similar to those of PD98059. The apoptosis rates of the cells treated with matrine and that of the cells treated with the pathway inhibitor PD98059 were much higher than that of the control cells, and most of the apoptotic cells were in the early stages of apoptosis. These experimental data suggest that matrine inhibits the proliferation of human OS cells mainly by inducing apoptosis.

## 4 Discussion

Prior to the 1970s, the primary strategy for treating OS was surgical tumor resection, which unfortunately resulted in a long-term survival rate of less than 20%. Notably, approximately 85% of deaths were attributed to distant metastasis of the tumor, predominantly to the lungs. Fortunately, with the introduction of neoadjuvant chemotherapy, the survival rate of OS patients has significantly increased to approximately 65%. However, based on recent large-scale randomized controlled trial results, the survival rate of OS patients appears to have plateaued at approximately 65%. This plateau might be associated with the lack of significant breakthroughs in our understanding of the key molecular mechanisms underlying OS; hence, efficacious therapeutic targets are still lacking. In light of this, there is an urgent need to thoroughly study the molecular pathogenesis of OS and to identify novel therapeutic targets with the aim of further enhancing patient survival rates and improving prognosis (15–17).

The application of traditional Chinese medicine in the treatment of OS has gradually attracted widespread attention in recent years. Among these agents, matrine, a quinolizine alkaloid extracted from the roots of traditional Chinese medicines such as *Sophora flavescens* and *Sophora sophora* flavescens, has been shown to perform various biological functions. Decades of studies have revealed a variety of biological activities of these compounds and their derivatives, including anticancer, anti-inflammatory, antiviral, analgesic, antifibrotic, antiparasitic and antibacterial activities. In particular, the anticancer activity of matrine has attracted the attention of researchers and the public (18, 19). However, the antitumor effect of matrine on OS remains unclear. Therefore, in the present study, we aimed to verify whether different concentrations of matrine can inhibit the proliferation of OS cells. Our findings showed that different concentrations of matrine had a dose-dependent effect on HOS cell proliferation. With the continuous development of modern medical technology and concepts, tumor treatment strategies have shifted from the simple inhibition of tumor cell proliferation to a more diversified path. Among these treatment approaches, promoting the apoptosis of tumor cells has gradually become the frontier of treatment research. Apoptosis is a finely regulated and programmed mode of cell death that plays a key role in maintaining cell homeostasis in the body. However, tumor cells often exhibit resistance to this natural apoptotic mechanism. Excessive proliferation and resistance to apoptosis are two major factors that contribute to tumor development and progression. Therefore, it is of great importance to study and develop drugs and treatments that can reactivate the apoptotic processes of tumor cells to improve the efficacy of antitumor therapy. In the present study, DAPI staining showed that the apoptosis rate of matrine-treated HOS cells was higher than that of the control cells. In addition, flow cytometry analysis showed that matrine effectively promoted the progression of tumor cells to the G1 phase of the cell cycle. The results showed that matrine inhibited cell proliferation and induced apoptosis in HOS cells and arrested the cell cycle at the G1 phase.

The biological characteristics of malignant tumors primarily include uncontrolled cell proliferation and continuous angiogenesis, which are closely associated with aberrations in cell cycle regulation (20). Malignant tumors exhibit significant migration and invasion during their progression. To effectively treat this disease, it is particularly critical to control the metastasis of tumor cells. In this study, the wound healing test provided us with an excellent platform for observing migration. According to the results, when the cells were treated with matrine, the wound area was markedly different from the untreated control, showing the effect of matrine on the behavior of the cells. To further confirm this observation, we then performed a Transwell assay. The results of this assay confirmed our observations, showing a significant decrease in the number of migrating and invading HOS cells in the matrine-treated group. Taken together, these findings provide strong evidence that matrine has a potential therapeutic effect by inhibiting the migration and invasion of cancer cells.

The occurrence of tumors is closely related to abnormalities in cell signaling pathways. Previous studies (21, 22) have shown that matrine can enhance the effect of doxorubicin through the STAT3 pathway and exert an anti-OS effect by inactivating the Akt pathway. In recent years, cellular signaling pathways have attracted significant attention in the field of cancer biology, with the MAPK/ERK pathway being particularly noteworthy (23, 24). This pathway plays a central role in various cellular physiological and pathological processes, including cell proliferation, differentiation, apoptosis, and stress responses. Our research indicates that matrine can inhibit the proliferation of osteosarcoma cells by suppressing the activation of the MAPK/ERK pathway. The MAPK/ERK pathway primarily consists of three cascading kinases, namely, MAPKKK, MAPKK, and MAPK. Notably, the molecular complexity of this pathway is further emphasized by its four main branches: p38, JNK, ERK1/2, and ERK5 (25, 26). Importantly, the ERK1/2 and ERK5 branches play a predominant role in cell proliferation, differentiation, and angiogenesis, while JNK and p38 are mainly related to stress responses and cell apoptosis (27). The diversity within the ERK family is also impressive, including members such as ERK1, ERK2, ERK3, ERK5, and ERK6 (28). Although ERK1/2 are often referred to as kinases that promote cell proliferation, their functions extend beyond this role, as they also mediate cell cycle arrest and (29). Moreover, ERK1 and ERK2 are clearly the most dominant members of the ERK family, and their abnormal activation is closely associated with malignancy in various cancers (30, 31). Studies on adrenal cortical tumors have shown the significant therapeutic potential of targeting the MAPK/ERK pathway. For instance, the MEK-MAPK-ERK pathway inhibitor PD184352 has been proven to significantly reduce the proliferation and steroidogenesis of the H295R adrenal cortical carcinoma cell line, providing valuable insights into new therapeutic approaches (32). Research on lung adenocarcinoma has also revealed the critical role of the MAPK/ERK pathway in regulating the proliferation of lung cancer stem cells, while the PI3K/AKT signaling pathway further promotes the proliferation of lung adenocarcinoma cells through its antiapoptotic mechanism (33). In summary, abnormalities in the signal transduction of the ERK pathway, such as overactivation or inhibition, may lead to excessive cell proliferation or prevent cell apoptosis, both of which are critical factors in tumor development (34, 35). A deeper understanding of the ERK/MAPK signaling pathway can not only reveal the molecular mechanisms of tumors but also aid in identifying novel targets for cancer therapy. Given the pivotal role of this pathway in various malignant tumors, there is a compelling case that therapeutic strategies that target this pathway will play a significant role in future anticancer strategies. Previous studies have shown that matrine can exert anti-OS effects by downregulating the ERK-NF-κB pathway (36), regulating Bax, Fas/Fasl and Bcl-2 (37), and inducing mitochondrial caspase-dependent apoptosis (38). (21) Studies have shown that matrine has significant effects on inhibiting growth and promoting apoptosis in HOS cells, and its anti-proliferation effects may be mainly dependent on the inhibition of AKT phosphorylation. Matrine can regulate AKT, glycogen synthesis kinase 3-β (GSK3-β), cyclin D1, p27Kip1 and Bax, which regulate the cell cycle and apoptosis. However, it can only increase the expression of Bax and inhibit the phosphorylation of AKT. In the present study, we used Western blotting to determine the expression levels of proteins involved in the MAPK/ERK signaling pathway. The results showed that matrine exerted effects that were similar to those of PD98059, a pathway inhibitor, leading to decreased expression and phosphorylation of Bcl-2. However, the expression and phosphorylation of Bax increased in a dose-dependent manner.

## 5 Conclusion

In conclusion, the present study showed that matrine inhibited cell proliferation, induced cell cycle arrest in the G1 phase, and promoted apoptosis, possibly by inhibiting the MAPK/ERK signaling pathway, in HOS cells. This may be related to the expression of Bax and Bcl-2. This study laid a theoretical foundation for the application of matrine in the treatment of OS. However, as a preliminary study, its limitations are also evident. We only analyzed one cell line in the *in vitro* experiments, and whether matrine exerts the same effect on other OS cell lines *in vitro* still needs further investigation. In the future, we will conduct experiments on other cell lines and other animal models to further explore the molecular mechanism. In addition, we need to further investigate the effects of matrine on other signaling pathways to more fully understand the mechanism underlying its anticancer effect. Overall, our study revealed a potential mechanism by which matrine inhibits the proliferation of OS cells, providing new evidence for its use as an anticancer agent. Future studies can further explore its potential application in the treatment of OS.

In our experiments, *in vivo* tests showed that matrine effectively inhibited tumor growth. By histological analysis, we observed a typical apoptotic cell morphology in the tumors of mice treated with matrine, which is consistent with the *in vitro* results. The immunohistochemistry results showed that matrine treatment decreased the expression of Bcl-2 and increased the expression of Bax. Although the subcutaneous transplantation model is convenient for drug delivery and tumor assessment, orthotopic transplantation is still a more ideal bone tumor model. Therefore, it is of great interest to use this model to further evaluate the therapeutic effect of matrine. Taken together, our study confirmed the ability of matrine to inhibit the growth of human OS cells *in vitro*. The possible mechanism involves the regulation of the Bcl-2/Bax ratio and the induction of OS cell apoptosis. This study provides a strong experimental basis for the use of matrine as a new strategy for treating OS, either alone or in combination with other drugs or monoclonal antibodies.

## Data availability statement

The original contributions presented in the study are included in the article/supplementary material. Further inquiries can be directed to the corresponding author.

## Ethics statement

Ethical approval was not required for the studies on animals in accordance with the local legislation and institutional requirements because only commercially available established cell lines were used.

## Author contributions

XH: Conceptualization, Methodology, Writing – original draft. JuZ: Writing – review & editing. SR: Writing – review & editing. ZL: Writing – review & editing. JiZ: Writing – review & editing. HC: Writing – review & editing.
